# Correction to “Risk factors and healing factors for pharyngocutaneous fistula after total laryngectomy for laryngeal cancer: An epidemiological study”

**DOI:** 10.1111/iwj.70008

**Published:** 2024-07-21

Tai Y , Liu F , Liu T , et al. Risk factors and healing factors for pharyngocutaneous fistula after total laryngectomy for laryngeal cancer: An epidemiological study. Int Wound J 2024;21(4):e14706. doi:10.1111/iwj.14706.38660912 PMC11044006

The correct corresponding authors should be Lingcao Ma and Yanzi Zang.

We apologize for this error.

